# Hypoxic Regulation of the Large-Conductance, Calcium and Voltage-Activated Potassium Channel, BK

**DOI:** 10.3389/fphys.2021.780206

**Published:** 2021-12-22

**Authors:** Sara V. Ochoa, Liliana Otero, Andres Felipe Aristizabal-Pachon, Fernando Hinostroza, Ingrid Carvacho, Yolima P. Torres

**Affiliations:** ^1^Departamento de Nutrición y Bioquímica, Facultad de Ciencias, Pontificia Universidad Javeriana, Bogotá, Colombia; ^2^Semillero de Investigación, Biofísica y Fisiología de Canales Iónicos, Pontificia Universidad Javeriana, Bogotá, Colombia; ^3^Center of Dental Research Dentistry Faculty, Pontificia Universidad Javeriana, Bogotá, Colombia; ^4^Department of Biology and Chemistry, Faculty of Basic Sciences, Universidad Católica del Maule, Talca, Chile; ^5^Centro de Investigación de Estudios Avanzados del Maule, CIEAM, Vicerrectoría de Investigación y Postgrado, Universidad Católica del Maule, Talca, Chile; ^6^Facultad de Ciencias de la Salud, Centro de Investigación en Neuropsicología y Neurociencias Cognitivas, Universidad Católica del Maule, Talca, Chile

**Keywords:** obstructive sleep apnea, BK channel, hypoxia, cardiovascular disease, MaxiK channel

## Abstract

Hypoxia is a condition characterized by a reduction of cellular oxygen levels derived from alterations in oxygen balance. Hypoxic events trigger changes in cell-signaling cascades, oxidative stress, activation of pro-inflammatory molecules, and growth factors, influencing the activity of various ion channel families and leading to diverse cardiovascular diseases such as myocardial infarction, ischemic stroke, and hypertension. The large-conductance, calcium and voltage-activated potassium channel (BK) has a central role in the mechanism of oxygen (O_2_) sensing and its activity has been related to the hypoxic response. BK channels are ubiquitously expressed, and they are composed by the pore-forming α subunit and the regulatory subunits β (β1–β4), γ (γ1–γ4), and LINGO1. The modification of biophysical properties of BK channels by β subunits underly a myriad of physiological function of these proteins. Hypoxia induces tissue-specific modifications of BK channel α and β subunits expression. Moreover, hypoxia modifies channel activation kinetics and voltage and/or calcium dependence. The reported effects on the BK channel properties are associated with events such as the increase of reactive oxygen species (ROS) production, increases of intracellular Calcium ([Ca^2+^]_i_), the regulation by Hypoxia-inducible factor 1α (HIF-1α), and the interaction with hemeproteins. Bronchial asthma, chronic obstructive pulmonary diseases (COPD), and obstructive sleep apnea (OSA), among others, can provoke hypoxia. Untreated OSA patients showed a decrease in BK-β1 subunit mRNA levels and high arterial tension. Treatment with continuous positive airway pressure (CPAP) upregulated β1 subunit mRNA level, decreased arterial pressures, and improved endothelial function coupled with a reduction in morbidity and mortality associated with OSA. These reports suggest that the BK channel has a role in the response involved in hypoxia-associated hypertension derived from OSA. Thus, this review aims to describe the mechanisms involved in the BK channel activation after a hypoxic stimulus and their relationship with disorders like OSA. A deep understanding of the molecular mechanism involved in hypoxic response may help in the therapeutic approaches to treat the pathological processes associated with diseases involving cellular hypoxia.

## Introduction

Calcium and voltage-activated potassium channels (BK channels) are ubiquitously expressed potassium (K^+^) channels involved in diverse physiological processes like the regulation of smooth muscle arterial tone (Latorre et al., 2017). The BK channel activity is modulated by different stimulus like membrane depolarization and intracellular Ca^2+^ concentration ([Ca^2+^]_i_). Functional variety of BK channels is associated with posttranscriptional and posttranslational modifications along with the association with accessories subunits called β (β1–β4), γ (γ1–γ4) and LINGO1 (Torres et al., 2007; Contreras et al., 2013; Dudem et al., 2020). BK channel activation causes membrane hyperpolarization, leading to a decrease in vasoconstriction and relaxation of the blood vessels. Modifications in channel activity increase the risk of pathologies associated with the vascular tone as hypertension (Amberg and Santana, 2003). Hypoxia, a condition where the level of oxygen is decreased, promotes diverse cellular responses as regulation of gene transcription, membrane depolarization, and changes in [Ca^2+^]_i_ (Prabhakar et al., 2001). Hypoxia modifies the function of diverse ion channels, including BK channels, through changes in the α/β subunits expression or changes in channel activity (McCartney et al., 2005). Diverse pathophysiological clinical conditions, including chronic obstructive pulmonary disease (COPD), asthma, and obstructive sleep apnea (OSA), are associated to hypoxia (Prabhakar et al., 2001). OSA is a disorder that induces chronic intermittent hypoxia by the predisposition of upper airway to fail during sleep (Sforza and Roche, 2016). Patients with OSA develop alterations including cognitive and cardiovascular diseases (Bradley and Floras, 2009; Benjafield et al., 2019). A myriad of mechanisms are involved in the effects of OSA in disease commencement and/or progression, and the function of BK channel has been proposed to play a critical role in the development of these pathologies (Navarro-Antolín et al., 2009; Caballero-Eraso et al., 2019). This review describes the diverse mechanisms involved in the modulation of the BK channel activity by hypoxia. In addition, we will discuss the role of the BK channel modulation in the progress of cardiovascular diseases in patients with OSA.

## Cellular and Molecular Mechanisms Associated With Hypoxia Responses

Oxygen (O_2_) is the central molecule for cellular respiration in aerobes. The level of O_2_ is controlled by specialized cellular sensors that are fundamental for maintaining its homeostasis. The fine regulation of O_2_ balance underlies cellular and overall physiological processes (Vjotosh, 2020). In humans, the rate of breathing regulates the availability of O_2_, which diffuses into the blood, binds to hemoglobin, and is distributed to all tissues (Schödel and Ratcliffe, 2019). In hypoxia, the level of O_2_ is insufficient for the maintenance of normal cellular function (Ziello et al., 2007). However, hypoxia can also be induced in normal physiological conditions during physical activity. The carotid body is the main peripheral organ sensing fluctuations in arterial O_2_, which is measured by changes in O_2_ tension. These variations trigger the reflex responses that aim to increase the pulmonary gas exchange, suppressing the hypoxia effects (Jonz et al., 2016). In addition, every cell needs mechanisms that ensure an adequate cell performance under diverse O_2_ concentrations and hypoxia (Schödel and Ratcliffe, 2019). For instance, in hypoxic episodes, different signals promote the enhancing of respiration to increase O_2_ levels in the lung by the constriction of lung vascular smooth muscle to improve blood flow and promote O_2_ delivery to different tissues. Moreover, metabolic pathways are modified to reduce oxygen consumption (Seta et al., 2004; Nakayama and Kataoka, 2019).

Hypoxia induces the activation of various mechanisms, including the modulation of ion channels, and the regulation of transcription factors that mediate the expression of several genes involved in the hypoxic response. Inhibition of voltage-gated K^+^ channels prompt membrane depolarization in excitable O_2_-sensing cells as glomus cells in the carotid body. Hypoxia also induces changes in [Ca^2+^]_i_ that conduces to the activation of protein kinases and phosphatases along with Ca^2+^-dependent signaling pathways involved in hypoxia gene expression (Seta et al., 2004). Therefore, [Ca^2+^]_i_ variations are the primary response of many cell types to hypoxia and have a significant role in the modulation of signaling pathways and gene expression.

Hypoxia-inducible factor (HIF) is a heterodimer comprising an oxygen-regulated α subunit and a constitutively expressed β subunit (Wang et al., 1995; Semenza, 2007). HIFs are transcription regulators that bind to specific DNA regions called hypoxia-responsive element (HRE), and are modulated by O_2_ level (West, 2017). In normoxia, HIF-1α is hydroxylated and binds the von Hippel-Lindau (VHL), a protein that targets HIF-1α for degradation via ubiquitin-proteasome (Tanimoto, 2000; Shimoda and Semenza, 2011). Diverse signaling pathways regulate HIF. Phosphorylation induced by PI3K/Protein kinase B (Akt) and protein kinase A (PKA) inhibits proteasomal degradation of HIF-1α. Additionally, mitogen-activated protein kinase/extracellular signal-regulated kinase (MAPK/ERK) promotes HIF-1α nuclear accumulation (Kietzmann et al., 2016; Xiao et al., 2017). In hypoxia, HIF-1α hydroxylation is inhibited and it is not targeted for degradation. Then, it translocates to the nucleus, where it dimerizes with HIF-1β. The dimer binds to specific HRE and regulates different target genes involved in the adaptation to hypoxia. These events increase the non-oxidative synthesis of ATP and prevent the excess of mitochondrial ROS generation, improving the O_2_-carrying capacity of blood and increasing the number of vessels irrigating the hypoxic tissues (Chandel et al., 1998; López-Barneo et al., 2001; Semenza, 2012). HIF regulates the expression of several hypoxic-associated genes, such as vascular endothelial growth factor (*VEGF)*, endothelial nitric oxide synthase *(eNOS)*, leptin *(LEP)*, LDL-receptor-related protein 1 *(LRP1)*, adrenomedullin *(ADM)*, epidermal growth factor *(EGF)*, metabolism [glucose transporter *(GLUT 1/3)*, hexokinase *(HK1/2)*, pyruvate dehydrogenase kinase 1 *(PDK1)*, pyruvate kinase M *(PKM)*, lactate dehydrogenase *(LDHA)*], cell proliferation [myelocytomatosis virus oncogene cellular homolog (c*-MYC)*, insulin-like growth factor 2 *(IGF2)*, DNA-binding protein inhibitor *(ID2)*, and inducible nitric oxide synthase *(iNOS)]*, transforming growth factor α *(TGFα)* and BCL2 Interacting Protein 3 *(BNIP3)* (Kelly et al., 2003; Manalo et al., 2005; Mole et al., 2009; Rey and Semenza, 2010; Shimoda and Semenza, 2011; West, 2017; Schödel and Ratcliffe, 2019; Sousa Fialho et al., 2019). HIF-1α also modulates redox signaling pathways in the heart. Particularly, during acute or chronic hypoxia where ROS is increased. The associated mechanism involves the up-regulation of the transcription levels of pro-oxidant molecules (Karar et al., 2007). Although some studies suggest that hypoxia decrease oxidative stress by increasing antioxidant activity, most of the studies demonstrate that hypoxia-induced ROS has a harmful effect (Aguilar et al., 2018).

Diverse cellular consequences of hypoxia have been reported. In addition to regulation of expression of genes by transcription factors, ion channel families have been described as direct effectors of the hypoxic response. A given ionic conductance, depending of the cells where they are expressed, can be inhibited or activated in response to hypoxia (López-Barneo et al., 2001). The increase in resistance of pulmonary arterioles, produced by the hypoxia-induced rise in arterial smooth muscle cell tone, is associated with the modulation of different ion channels activity. Transient Receptor Potential (TRP) channels, L-type Ca^2+^ channels, K_v_ channels as BK, and TWIK-related tandem pore domain acid-sensitive K^+^ channel (TASK)-type are important to set the resting membrane potential and modulate membrane depolarization (Wang J. et al., 2006; Weissmann et al., 2006; Sommer et al., 2008; Whitman et al., 2008; Jonz et al., 2016). In the next sections, we will describe the direct effect of hypoxia on the activity of the large conductance, Ca^2+^ -activated K^+^ (BK), and their association with pathological conditions underpinning cardiovascular diseases.

## Hypoxia and Cardiovascular Diseases

Hypoxia can relieve or intensify the severity of risk factors associated with cardiovascular diseases (CVD) (Ullah and Wu, 2021). Chronic hypoxia is associated with the generation of pathological conditions, such as ischemic ventricular arrhythmias, cardiomyocyte and cardiac death, pulmonary hypertension, and liver fibrosis (Madjdpour et al., 2003; Mesarwi et al., 2016; Moczydlowska et al., 2017; Morand et al., 2018; Corrado and Fontana, 2020). On the contrary, acute hypoxia has been demonstrated to have a protecting role in acute liver and kidney diseases, and in myocardial ischemia (Schellinger et al., 2017; Shu et al., 2019; Gao et al., 2020; Ullah and Wu, 2021).

Ischemic heart disease, heart failure, or OSA, among other diseases, can induce hypoxic conditions. Hypoxia induces activation of HIF and their associated signaling pathways (Corrado and Fontana, 2020). OSA is a disorder where recurrent pauses of ventilation during the sleep conduce to upper airway obstruction, intermittent hypoxemia, and the increase in respiratory effort (Chen et al., 2021; Diamond and Ismail, 2021; Mochol et al., 2021). OSA has a high prevalence depending on age and ethnic group, and it is a major public health issue due to its effect on work performance and productivity (De Luca Canto et al., 2015). Benjafield et al. (2019) estimated that OSA affects almost 1 billion people in the world (Benjafield et al., 2019). There are reports about the modification of the equilibrium pro/antioxidant as patients with OSA show a rise in the superoxide anion level, lipid peroxidation, and diminished antioxidant mechanism (Arnaud et al., 2020). The latter is associated with recurrences of hypoxia-reoxygenation cycles and production of superoxide anion, which promotes the stabilization of HIF-1α through the activation of phospholipase C pathway (Belaidi et al., 2016; Farías et al., 2016). OSA adversely alters the cardiovascular structure, inducing changes such as endothelial dysfunction. Additionally, OSA is reported as an independent risk factor for initiation or progression of cardiovascular diseases like hypertension, heart failure, ischemic heart disease, and stroke (Marin et al., 2005; Bradley and Floras, 2009; Wang et al., 2013; Orrù et al., 2020; Chen et al., 2021; O’Donnell et al., 2021). The treatment of OSA includes continuous positive airway pressure (CPAP) and mandibular advancement devices (MAD), but there is no effective drug therapy for OSA. CPAP and MAD keep the upper airway open during sleep. It has been suggested that CPAP and MAD therapies attenuate some cardiovascular diseases including atherosclerosis, hypertension, heart failure, and cardiac arrhythmias (Barnes et al., 2004; Gotsopoulos et al., 2004; Bradley and Floras, 2009; Marklund et al., 2019; Yamamoto et al., 2019; Chen et al., 2021; O’Donnell et al., 2021).

## BK Channel Structure, Localization, and Modulation

Large conductance, Ca^2+^ -activated K^+^ (BK) channels are K^+^ channels ubiquitously expressed in mammalian cells. They are involved in different physiological processes like the regulation of smooth muscle arterial tone and neurotransmitter release, among others (Latorre et al., 2017). BK channels are tetramers where each subunit is constituted by the pore-forming α subunit formed by seven transmembrane domains (S0-S6), being the NH_2_ termini exposed to the extracellular side, and the COOH termini facing the intracellular. In that region, there are two regulators of conductance of K^+^ domains named RCK1 and RCK2 (Meera et al., 1997; Jiang et al., 2001; Torres et al., 2007; Wu et al., 2010; Pantazis and Olcese, 2016). BK α subunit has a modular organization with the pore region comprised between the S5-S6 region and the voltage sensor located in S0-S4 transmembrane domains (Meera et al., 1997; Díaz et al., 1998). The presence of multiple Ca^2+^ -binding sites confers to the channel sensitivity to a vast Ca^2+^ concentration range (0.1–100 μM) (Bao et al., 2004; Latorre and Brauchi, 2006). The Ca^2+^ -binding site called Ca^2+^ bowl, located in the RCK2, acts as a high affinity Ca^2+^ binding site (Wu et al., 2010). Functional diversity of the channel is generated by different BKα splice variants, posttranslational modifications, and the association with auxiliary β, γ, and LINGO1 subunits (Contreras et al., 2013; Pantazis and Olcese, 2016; Dudem et al., 2020).

Alternative splicing at the C terminus region produces diverse splice variants of the BK channel including ZERO (channels without the STREX insert) and Stress-Regulated Exon (STREX), which have cellular differential expression and tissue distribution (Zarei et al., 2001; Chen et al., 2005; Contreras et al., 2013). STREX variant is a highly conserved motif within an alternatively spliced cysteine-rich insert. This feature confers to the channel sensitivity to high Ca^2+^ and hypoxia (Shipston et al., 1999). Hypoxia induces a reduction in the BK channel open probability (Po) by a Ca^2+^ -dependent shift to the right in the voltage activation. Residues C23, S24, and C25 are critical for the hypoxic response in the STREX variant (McCartney et al., 2005). ZERO is a cAMP-sensitive splice variant that induces a lower Ca^2+^ sensitivity, compared with the STREX variant (Shipston et al., 1999; Friis et al., 2003).

The physiological function of BK channels varies depending on both cell type and cellular localization. For example, in sinoatrial node (SAN) cells and vascular smooth muscle, BK channels are expressed in the plasma membrane (Leblanc et al., 1994; Gollasch et al., 1996; Lai et al., 2014), whereas in cardiomyocytes, BK channels are localized at the inner mitochondrial membrane (IMM) (Singh et al., 2013; Goswami et al., 2019).

Sinoatrial node (SAN) cells act as a pacemaker of the cardiac conduction system, and thus, determine the heart rate (Mangoni and Nargeot, 2008). The functional expression of BK channels in SAN cells have been demonstrated using paxilline, a BK channel inhibitor. Paxilline administration in mice reduces the heart rate in wild-type (WT) but not in BK channel α subunit knock-out animals (*kcnma1^–/–^*). Specifically, paxilline decreases action potential firing in SAN cells. Immunocytochemistry revealed that BK channels are expressed in the plasma membrane and partially overlapped with the hyperpolarization activated cyclic nucleotide gated K^+^ channel 4 (HCN4) (Lai et al., 2014). BK channels are also expressed in human coronary artery vascular smooth muscle cells (Gollasch et al., 1996) and in rabbits’ coronary myocytes. In these cells, their role is to maintain the resting membrane potential (Leblanc et al., 1994).

Calcium and voltage-activated potassium channels (BK) are also expressed in the IMM but not in the plasma membrane in mice cardiomyocytes. In this cell type, a splice variant from the plasma membrane *Kcnma1* gene is expressed in the IMM. This variant targets the mitochondria through its C-termini (Singh et al., 2013), which differs from other proteins that target mitochondria through its N-termini (Chacinska et al., 2009; Li M. et al., 2010). In mitochondria, the BK channels play an essential role in protecting the heart from ischemic insult (see section “Mitochondrial BK Channel Participate in Hypoxic Protection”). Moreover, in cardiomyocytes of infant rabbits, mitochondrial BK channels also protect the heart against ischemia (Shi et al., 2007).

The regulatory β1-subunit of BK channels is also expressed in cardiomyocytes, and it colocalizes with BK channels in the IMM. Furthermore, β1-subunit functionally interacts with BK channels increasing the Po. Additionally, β1-subunit increases the localization of BK channels at the IMM (Balderas et al., 2019).

### Auxiliary Subunits Modulate the BK Channel Properties

**FIGURE 1:**
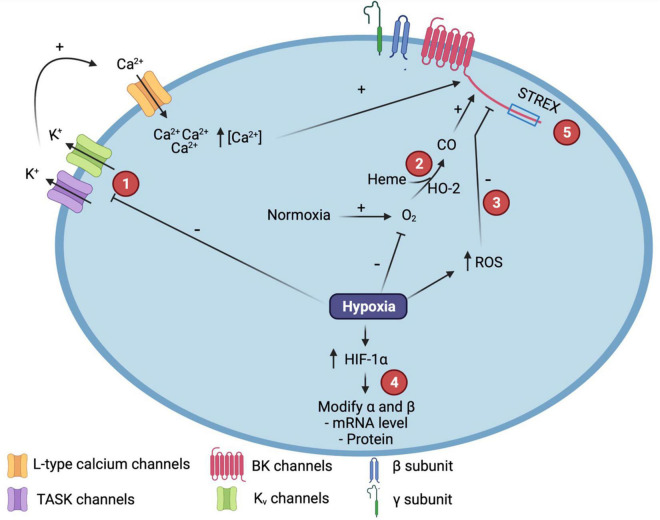
Molecular mechanisms involved in the modulation of Calcium and voltage-activated potassium (BK) channel function by hypoxia. (1) Hypoxia promotes TWIK-related tandem pore domain acid-sensitive K^+^ channel (TASK) or Kv channels inhibition that induces membrane depolarization, activation of L-type voltage-dependent calcium channels and the increase in intracelular calcium concentration, underpining BK channel activation. (2) In normoxia, hemeoxigenase (HO-2) catalyzes Heme degradation to produce carbon monoxide (CO), increasing the open probability (Po) of the BK channel. (3) ROS production increased after hypoxia induces the decreasing of BK channel Po. (4) After hypoxia, HIF-1α is increased and modulates BK subunits expression through the interaction with hypoxia-response elements (HRE). (5) STREX variant confers to BK channel sensitivity to hypoxia. ROS, Reactive Oxygen Species; HIF, Hypoxia Inducible Factor. Created with BioRender.com.

Differential expression of BK auxiliary subunits confers to the channel functional diversity (Figure 1). In the smooth muscle, the α subunit is co-expressed with the β1 subunit, which prompts membrane hyperpolarization and vasorelaxation, decreasing the risk of pathologies associated with vascular tone regulation (Amberg and Santana, 2003). The β1-induced effects are associated with the improvement of apparent sensitivity to Ca^2+^ and slowing down the activation and deactivation kinetics. These effects are elicited by changes of the allosteric coupling between gating and the Ca^2+^ binding sites along with the reduction of the voltage dependence of the voltage sensor activation process (Nimigean and Magleby, 2000; Orio and Latorre, 2005; Contreras et al., 2012). β2 subunits also increase the apparent Ca^2+^ sensitivity and slowed the gating kinetics, associated with an increase in the allosteric coupling factors (Orio and Latorre, 2005). The β3 subunit has four members (β3a, β3b, β3c, and β3d). The most studied members are β3a and β3b, which induce BK channel inactivation (Brenner et al., 2000a; Uebele et al., 2000; Gonzalez-Perez and Lingle, 2019). It has been reported that β4 subunit slows the gating kinetics, reduces the apparent voltage-sensitive of the channel activation, and modifies the apparent Ca^2+^ sensitivity in two ways: by the inhibition of the channel activity in low [Ca^2+^]_i_ and by the increase of the channel activity in high [Ca^2+^]_i_. These effects are associated with the stabilization of the active conformation of the voltage sensor, and a reduction in the number of the gating charges per sensor (Brenner et al., 2000a; Wang B. et al., 2006; Contreras et al., 2012).

γ1 (LRRC26), γ2 (LRRC52), γ3 (LRRC55), and γ4 (LRRC38) subunits display tissue-specific expression and function (Yan and Aldrich, 2012; Evanson et al., 2014). They produce a significant modification in the voltage dependence of BK channel activation, recognized as a shift to negative potentials of ∼140, 100, 50, and 20 mV in the presence of γ1 (LRRC26), γ2 (LRRC52), γ3 (LRRC55), and γ4 (LRRC38), respectively (Yan and Aldrich, 2012; Zhang and Yan, 2014). The increase in voltage sensitivity induced by γ1 has been associated with vasodilation observed in arterial smooth muscle cells (Evanson et al., 2014). Moreover, knocking down the expression of the γ1 subunit contributes to vasoconstriction (Yan and Aldrich, 2012). γ1 subunit also induces the acceleration of the activation kinetics, while the deactivation kinetics become slower. It has been proposed that γ2, γ3, and γ4 subunits can modify the apparent Ca^2+^ sensitivity (Latorre et al., 2017). LINGO1 is an accessory subunit of BK channels that induces a rapid inactivation of the channel, slowing down the deactivation process and shifting their activation to negative potentials (Dudem et al., 2020).

### Posttranslational Regulation of BK Channel

Palmitoylation is a post-translational modification associated with regulation of the BK channel plasma membrane localization without variations in single-channel conductance or the Ca^2+^ and voltage sensitivity. BK channel palmitoylation occurs in the STREX insert and in the residues C53, C54, and C56, located in the intracellular loop S0-S1 (Jeffries et al., 2010; Zhou et al., 2012).

On the other hand, BK α or β subunits undergo reversible posttranslational modifications as protein phosphorylation. These control BK channel activity. Protein kinase A (PKA), Ca^2+^—and diacylglycerol-activated protein kinase C (PKC), cyclic guanosine monophosphate (cGMP) -activated protein kinase G (PKG), and adenosine monophosphate (AMP)-activated protein kinase (AMPK) have been reported to phosphorylate BK channel α subunit in serine, threonine, and tyrosine residues. The phosphorylation in the BK channel activity is depending on the channel variant. For instance, PKA stimulates ZERO variant activation but induces STREX splice variant inhibition. The final effects of phosphorylation are tissue-depending, like inducing BK channel activation in smooth muscle but inhibit the channel activity in pituitary cells (Contreras et al., 2013; Kyle and Braun, 2014; Shipston and Tian, 2016).

### Modulation of BK Channel Activity by Endogenous Molecules

Diverse endogenous molecules, including heme, carbon monoxide (CO), and reactive oxygen/nitrogen species, have been reported to activate BK channels (Hou et al., 2009; Kyle and Braun, 2014). The free intracellular heme binds to a region in the RCK1 and RCK2 segments and decrease or increase the BK channel Po at positive or negative voltages, respectively. These effects are modulated by the action of heme oxygenase enzyme (HO) by regulating the heme group degradation (Horrigan et al., 2005; Muñoz-Sánchez and Chánez-Cárdenas, 2014). Electrophysiological assays demonstrated that CO increase the Po of the channel in cell-free membrane patches, suggesting channel modulation by CO direct binding in RCK1 domain (Jaggar et al., 2002; Hou et al., 2008; Williams et al., 2008).

Calcium and voltage-activated potassium (BK) channels redox modulation has been widely described (Hou et al., 2009). H_2_O_2_ decreases the Po through amino acid modifications. These are reversed by the addition of reducing agents as glutathione (GSH) or dithiothreitol (DTT). C433 and C911, located in the RCK1 and COOH terminus, respectively, have been reported to contribute to the sensitivity of the BK channel to reactive species (DiChiara and Reinhart, 1997; Tang et al., 2001, 2004; Soto et al., 2002; Brakemeier et al., 2003). STREX variant has additional cysteine residues that increase the inhibitory effect of oxidation in the channel (Erxleben et al., 2002). O_2_^–^, NO, and peroxynitrite are reactive molecules also affecting the BK channel activity (Hou et al., 2009). Finally, fatty acids, phospholipids, steroids, and other lipid metabolites can also modulate BK channel properties (Hou et al., 2009). In another way, it has been reported that decreases in intracellular pH increase BK channel activation by inducing a shift to the left in the voltage-dependence (Avdonin et al., 2003; Hou et al., 2009; Kyle and Braun, 2014).

As was described, the structural properties of the BK channels play different important physiological roles. In addition, both BK channel expression and its activity are regulated by various mechanisms which confer functional diversity to the channel. In the next section, we will describe the reported mechanism involved in the modulation of the BK channel by hypoxia and the possible physiological impact of that modulation.

## BK Channel Modulation in Hypoxia

Calcium and voltage-activated potassium (BK) channel has been described as an oxygen-sensor, being one of the most important intermediaries in the hypoxic response of different tissues (McCartney et al., 2005). Hypoxic-mediated BK channel response is diverse, i.e., it is increased in some tissues while reduced in others. The modifications in the BK channel activity induced by hypoxia can be related to maintain the hypoxia response or decrease the hypoxic effects that induce cell damage such as changes in [Ca^2+^]_i_. These effects are mediated by diverse mechanisms including oxidative stress (Liu et al., 1999), heme protein interaction (Hoshi and Lahiri, 2004), the gene transcription regulation mediated by HIF-1α (Resnik et al., 2006; Ahn et al., 2012; Tao et al., 2015), and splice-variant-specific pathways (McCartney et al., 2005). In this section, we will describe the principal mechanisms involved in the BK channel response to hypoxia. Further, we revise the effects in both channel activity and BK subunits expression induced by decreased in O_2_ level (Figure 1).

### Hypoxic-Induced Modulation of the BK Channel Activity

It has been previously reported that hypoxia promotes a rise in the BK channel activity through membrane depolarization, and increases in [Ca^2+^]_i_. Hypoxia promotes membrane depolarization by different mechanisms including the inhibition of K^+^ channels as K_v_1.2 and K_v_1.5 (Seta et al., 2004). Blocking K_v_ channels with the antagonist tetraethylammonium (TEA) evoked a similar response as hypoxia in glomus cells (Pardal et al., 2000; López-Barneo et al., 2001; Wang and Kim, 2018). TASK channels expressed in glomus cells are also hypoxic-inhibited channels. It has been reported that a decrease in cytosolic Mg-ATP or phosphorylation via AMP-activated kinases, among others, are events that promote TASK inhibition leading to membrane depolarization, consequent activation of L-type voltage-dependent Ca^2+^ channels, and rising in [Ca^2+^]_i_, and consequently increasing BK channel Po (Buckler, 2015; O’Donohoe et al., 2018).

Hippocampal neurons exposed to hypoxia for 6 h exhibited a significant rise in BK channel Po and unitary conductance after 6 h of reoxygenation. The normal parameters in channel activity were recovered after 24 h of reoxygenation. These results were associated with an increase in [Ca^2+^]_i_ without changes in the BK α subunit expression (Chen et al., 2013; Wang and Kim, 2018). Some authors have proposed that BK channels are active at rest, and that hypoxia inhibits their activity (Peers and Wyatt, 2007). On the contrary, some reports suggest that BK channels are closed in normoxia while hypoxia induces the channel activation to diminish the hypoxia-induced cell damage (Pardal et al., 2000; Gomez-Niño et al., 2009). The overall effect of hypoxia-induced BK channel activity on cell survival depends on specific factors present in a given cell type or tissue. For instance, the activation of the BK channel after hypoxia/reoxygenation induces hippocampal neuronal apoptosis. Meanwhile the hyperpolarization induced in glomus cells by BK channel activation constraints the rise in [Ca^2+^]_i_, and, therefore, limits the hypoxic response (Chen et al., 2013; Wang and Kim, 2018).

### Changes in the Expression of the BK Channel Subunits Induced by Hypoxia

It is known that BK β subunits expression contributes to the molecular diversity of BK channels (Torres et al., 2014). An important role for BK β1 subunit in regulating vascular tone was demonstrated by systemic hypertension in mice carrying a deletion of the *Kcnmb1*, the gene encoding for β1 subunit (Brenner et al., 2000b; Chang et al., 2006). Additionally, the association between severity of asthma and a loss-of-function polymorphism in *Kcnmb1*, suggests a role of this subunit in modulating smooth muscle cells tone of airway in humans (Seibold et al., 2008). Different reports show that hypoxic response can also be modulated by differential expression of β subunits. In ovine pulmonary artery smooth muscle cells (PASMC), primary cultures, and ovine cerebral artery smooth muscle, hypoxia induced a rise in mRNA and protein expression level of both BKα and β1 subunits. Similar results were observed in rats that have been maintained in hypobaric hypoxic chambers for 3 weeks where mRNA levels of BKα and β1 subunits increased approximately threefold and twofold, respectively (Resnik et al., 2006; Tao et al., 2015).

The rise in the β1 subunit expression enhances the BK channel apparent sensitivity to [Ca^2+^]_i_ and the current density, leading to hyperpolarization, subsequent closing of voltage-gated Ca^2+^ channels, and decreasing the [Ca^2+^]_i_ (Torres et al., 2007; Castillo et al., 2015). The increase in BK channel activity may offer an adequate brain O_2_ level when there is a lower arterial O_2_ concentration, as it was reported in ovine artery smooth muscle (Tao et al., 2015).

Reported changes in the BK subunits expression are suggested to occur via post-translational modification by phosphorylation/dephosphorylation and by transcriptional regulation through the interaction with HIF-1α (Resnik et al., 2006; Ahn et al., 2012; Tao et al., 2015). After hypoxic treatment of hPASMC, it was observed an increase in the expression of both HIF-1α and KCNMB1 and knocking down HIF-1α avoided the hypoxia-induced KCNMB1 expression. In addition, it was demonstrated that human KCNMB1 promoter has HREs that are critical for HIF-1α binding and hypoxic-modulation of KCNMB1 expression (Ahn et al., 2012). *Kcnmb1^–/–^* mice showed higher right ventricular systolic pressure after hypoxic stimulus, compared with WT mice. These experiments demonstrated the importance of the BKβ1 subunit in the modulation of the pulmonary vascular response to chronic and acute hypoxia, and suggests a connection between BKβ1 expression and HIF-1α activity in the regulation of the tone in the microcirculation (Barnes et al., 2018).

Besides the increase in the BKβ subunit expression, it was reported that chronic hypoxia enhanced α/β colocalization at the plasma membrane without changes in mRNA expression, suggesting post-transcriptional regulation of the BK β subunit (Hartness et al., 2003). From these results, it has been proposed that hypoxic events originated from diverse cardiorespiratory diseases, conduct to adaptative cellular responses, including changes in the BK subunit expression or increase in functional α/β complex at the plasma membrane (Hartness et al., 2003).

In some cells/tissues a decrease in β subunit expression has also been reported after hypoxic stimuli. Rat myocytes exposed to prenatal hypoxia showed a reduction in the channel voltage and Ca^2+^ -sensitivity, associated with a drop in the BK β1 subunit mRNA and protein expression. These changes were without variations in the BKα subunit expression (Liu et al., 2018a,b). All these findings suggest that BK channel modulation induced by hypoxia is cell specific. BK channels expressed in rat vascular smooth muscle cells are functionally less active, decreasing the vasorelaxant effect of the channel and leading to high blood pressure, vascular dysfunction, and cardiovascular alterations (Lewis et al., 2002; Navarro-Antolín et al., 2005; Liu et al., 2018a). However, in ovine PASMC primary cultures and ovine cerebral smooth muscle, hypoxia induced a rise in mRNA and protein expression level of both BKα and β1 subunits.

The BKβ4 subunit expression is also modulated by hypoxia. In podocytes, an increase in BK β4 mRNA and protein levels were observed after chronic hypoxia without modifications in the expression of the BK α subunit. The higher expression of the β4 subunit decreases the BK channel activity by a shift in the voltage-dependent activation toward depolarized voltages, and a significant increase in the time constant for channel activation. In conclusion, Zhang et al. (2012), suggested that reducing BK channel activity promotes podocyte depolarization, leading to a decrease in Ca^2+^ influx through TRPC6 channels. The consequent change in [Ca^2+^]_i_ modifies Ca^2+^-dependent signaling pathways associated with hypoxic response (Zhang et al., 2012).

In addition to increases in BK β subunit expression, a rise in mRNA from BK channel α subunit, and changes in the protein distribution were positively associated with the extent of the hypoxic response in pulmonary arteria. That response correlates with structural changes as alterations in the intimal thickening, suggesting an adaptative response in patients with the COPD to attenuate hypoxic pulmonary vasoconstriction (Peinado et al., 2008).

### Mechanism Mediating BK Channel Response to Hypoxia

Calcium and voltage-activated potassium (BK) channel activity is modulated by additional mechanisms, including heme oxygenase-2 (HO-2), ROS regulation, and STREX-associated interaction (Hoshi and Lahiri, 2004; Navarro-Antolín et al., 2005; Figure 1). In mammalian cells, the enzyme HO-2 is implicated in the degradation of the heme group to produce biliverdin, iron, and carbon monoxide (CO). HO-2 function has been associated to O_2_ sensing and hypoxic response through the regulation of the BK channel activity (Hoshi and Lahiri, 2004; Ortega-Sáenz et al., 2006; Muñoz-Sánchez and Chánez-Cárdenas, 2014). The mechanism involves the BK channel activation by CO and the physical interaction of HO-2 with the channel. It has been reported that knocking down of HO-2 induces a decrease in the expression of BK α subunit. Hypoxia can induce a redox dysregulation that prompts the HO-2 deficiency, decreases the CO levels, and reduces the channel activity (Naik and Walker, 2003; Williams et al., 2004; Ortega-Sáenz et al., 2006). Co-immunoprecipitation with HO-2 in carotid body cells was only observed when channels were expressing both α and β subunit (Riesco-Fagundo et al., 2001; Williams et al., 2004; Ortega-Sáenz et al., 2006). On the contrary, a weak interaction between HO-2 and BK channel was observed in pulmonary arterial smooth muscle. Furthermore, in mouse lines deficient in either HO-2 or BKα subunit it was not observed any role of these proteins in the hypoxic effects (Roth et al., 2009), suggesting that hypoxic-modulated interaction and function of HO-2 and BK channels could be tissue-specific and the interaction HO-2/BK channel could not be considered as a universal O_2_ sensor. Currently, there are not reports related to the role of γ or LINGO1 subunits in the modulation of BK channel function by hypoxia.

Another mechanism involved in the BK channel modulation is associated with redox regulation of BK channel binding of the heme group. In normoxia, the heme group has a low affinity to the channel in contrast to the high affinity that shows for HO-2. That event induces heme degradation and an increase in CO, promoting channel activation. On the contrary, in hypoxia, HO-2 has a lower affinity for the heme group, generating an increase in heme concentration and decreasing CO production. Both stimuli reduce the channel activity (Tang et al., 2003; Yi and Ragsdale, 2007; Ragsdale and Yi, 2011) (Figure 1).

BK channel is also modulated through cAMP and GMP-dependent protein kinase (PKG) by specific phosphorylation of multiples Ser residues in the C terminus of the α subunit. PKC-dependent phosphorylation shifts the voltage-dependence to more negative potentials, increasing BK channel voltage-sensitivity (Zhou et al., 2001; Kyle et al., 2013). In middle cerebral arteries, long-term hypoxia affected the PKG-modulation of the BK channel activity. It was found that hypoxia reduces the BKα subunit expression and decreases the association of PKG with the BK channel, inhibiting the channel activation and the consequent vasorelaxation (Thorpe et al., 2017).

On the other hand, hypoxia and endogenous H_2_S induce similar inhibition of the BK channels in the carotid body. The inhibition mediated by H_2_S endogenous production demonstrated the involvement of that molecule in the hypoxic-modulated BK channel activity, and suggest it as sensor for hypoxia in vasculature (Li Q. et al., 2010). A contrary effect was reported in vascular smooth muscle, where H_2_S has a regulating function of myogenic tone through BK channel activation (Jackson-Weaver et al., 2011). These results demonstrated that BK channel modulation depends on the cell type, however, more studies are necessary to establish the effect of H_2_S in hypoxic modulated BK channel activity.

Calcium and voltage-activated potassium (BK) channel inhibition, induced by low O_2_ in neocortical neurons, is mediated by variations in the cellular redox potential, and depends on cytosolic factors, demonstrating the channel regulation by oxidative stress (Liu et al., 1999). Similarly, in rat CA1 hippocampal neurons, hypoxia induced a decrease in the BK channel Po due to a reduced mean channel open time and an increased closed time. Channel activity was restored after treatment with oxidizing agents, suggesting that hypoxia decreases cellular oxidation potential in CA1 neurons (Gao and Fung, 2002). Furthermore, hypoxia stimulates the production of reactive species ROS and reactive nitrogen species (RNS), and increases oxidative stress (Fresquet et al., 2006; Hu et al., 2016). In smooth muscle from sheep, uterine artery was reported a significant effect of stress oxidative in the inhibition of BK channel activity by hypoxia. These changes were showed to be mediated by downregulation of the BK β1 subunit (Zhu et al., 2014; Hu et al., 2016). The authors reported that the treatment with the antioxidant N-acetylcysteine (NAC) eliminates the hypoxic-mediated inhibition of the BK channel’s current density, reestablished β1 expression, and restored the arterial tone. After NAC treatment, there were no differences in relaxation induced by the BK channel agonist NS1619 in normoxic and hypoxic animals. In addition, the antioxidant treatment also rescues the hormonal steroid effect induced on the BK channel activity. The proposed mechanism involves post-translational modifications induced by ROS and KCNMB1 gene repression (Zhu et al., 2014; Hu et al., 2016).

Reactive oxygen species (ROS) effect has been reported in cardiovascular diseases. Moreover, it has been proposed that oxidative stress induced by ROS impairs the BK channel activation by changing the cysteine 911 (C911), located in RCK2 near to the Ca^2+^ bowl. It was shown that adding H_2_O_2_ to the intracellular side induced the BKα + β1 channel inhibition through a decrease in Po and Ca^2+^ sensitivity without modification of the unitary conductance. Authors suggest that observed functional properties of the BK channel after oxidative stress are comparable to the found in absence of β1 subunit (Soto et al., 2002; Tang et al., 2004; Tano and Gollasch, 2014).

Hypoxia promotes a decrease in the modulatory effect that tamoxifen and steroid hormone induces on the BK channel activity, which was associated with the diminished expression of the BK β1 subunit (Navarro-Antolín et al., 2005; Liu et al., 2018a). Recently, we reported that changes in cholesterol concentration reduces the effects of 17β-Estradiol (E2) in the BK channel activity. However, that effect was not induced by changes in the expression of the BK β1 subunit (Granados et al., 2021). Considering that hypoxia reduces the membrane cholesterol concentration, we suggested that reported changes in the modulation of Tamoxifen and E2 after hypoxia could also be related to changes in membrane cholesterol concentration. However, it is necessary to carry on more experiments to unveil the complete mechanisms associated with that effect (Zhang et al., 2018).

McCartney et al. (2005) reported that BK channel sensitivity to hypoxia is splice-variant-specific, conferred by the stress-regulated exon (STREX) (Figure 1). The hypoxic inhibition induced in the STREX variant is Ca^2+^-sensitive with no effect on single-channel conductance nor the voltage sensitivity. The hypoxic inhibition required Serine S24 and Cysteines C23 and C24, suggesting an important role of these residues in the hypoxic response. The ZERO variant was non-sensitive to hypoxia (McCartney et al., 2005; Tano and Gollasch, 2014).

### Mitochondrial BK Channel Participate in Hypoxic Protection

In addition to plasma membrane BK channels, there are diverse reports about the involvement of mitochondrial BK channels (mitoBK_Ca_) in the hypoxic response and protection against ischemic injury (Xu et al., 2002; Shi et al., 2007; Goswami et al., 2019). mitoBK_Ca_ is expressed in adult cardiomyocytes and it is encoded by the splice variant of the *Kcnma1* gene DEC, which has an insert of 50 amino acids in the C-terminal region that is responsible for targeting the channel to mitochondria (Singh et al., 2013; Szteyn and Singh, 2020). mitoBK_Ca_ has been reported to have similar properties to BK channels expressed in the plasma membrane (Xu et al., 2002; Balderas et al., 2019). These channels induce a cardioprotective mechanism that involves the decrease of ROS production and the depolarization of the mitochondria matrix by K^+^ flux and the reduction of Ca^2+^ overloading during ischemia and reperfusion (Xu et al., 2002; Stowe et al., 2006; Shi et al., 2007; Borchert et al., 2011; Goswami et al., 2019). Interestingly, the reported effect was observed only in the normoxic hearts but not in chronic hypoxic hearts. Considering that BKα subunit expression was not changed, it is suggested that the effect was associated with a significant reduction in mitoBK_*Ca*_ channel activity, probably via decreasing in Ca^2+^ sensitivity (Riesco-Fagundo et al., 2001; Shi et al., 2007). In addition to BKα subunit, it has been demonstrated the mitochondrial expression of β1 subunit in cardiac tissue and myocytes, where it is critical to mediate cardioprotective response (Wang et al., 2008; Borchert et al., 2011; Balderas et al., 2019). These effects are not related to changes in the β1 subunit expression but are associated with activation of the channel possibly induced by modifications in glycosylation level of β1 subunit. However, more assays are necessary to prove the suggested hypothesis (Wang et al., 2008; Borchert et al., 2011).

Experiments using BK β1 KO mice demonstrated that mitoBK_Ca_ channel activity in mitochondria was negligible when BK β1 subunit was absent, suggesting a reduction in the number of channels in mitochondria. Moreover, it was proposed a role of β1 in targeting BK channel to mitochondria. Activity of mitoBK_Ca_ was associated with Ca^2+^ homeostasis in cardiac cells (Balderas et al., 2019). The presence of a second population of mitoBK_Ca_ that activates at more depolarizing potentials suggests that BK can be associated with other regulatory subunits to produce channels with different voltage-sensitivity (Balderas et al., 2019). In addition, Frankenreiter et al. (2017), reported a positive regulation of mitoBK_Ca_ by cGMP, which regulates ROS homeostasis in oxidatively stressed cardiomyocyte mitochondria and induces a significant increase in channel Po. That effect is associated with cardioprotective properties in models of ischemia/reperfusion injury possible through cGMP/cGKI (cGMP-dependent protein kinase type I) pathway (Frankenreiter et al., 2017).

Hypoxic modulation of the BK channel depends on cells or tissue. In some cells, hypoxic stimuli inhibit the BK channel activity. Meanwhile, in other cells, hypoxia promotes channel activation. Modulation of the channel activity has an important role in the hypoxic response, as it has been proposed to act as an “emergency brake.” BK channel response to hypoxia has been associated with the development of diverse pathologies such as preeclampsia (Hu et al., 2012; Zhu et al., 2013) and neuronal injury (Tjong et al., 2008). In addition, there are several reports about the role of BK channel in hypoxic-modulated cardiovascular diseases, like pulmonary artery hypertension derived from COPD, chronic inhalation of CO, or OSA (Bonnet et al., 2003; Dubuis et al., 2005; Peinado et al., 2008; Navarro-Antolín et al., 2009; Roth et al., 2009; Ahn et al., 2012; Barnes et al., 2018; Liu et al., 2018b; Li et al., 2019). Moreover, it has been proposed that BK channel openers might be used in stroke, epilepsy, asthma, and hypertension (Kirby et al., 2013).

## BK Channel Activity in the Hypoxia Response Derived From Obstructive Sleep Apnea

Intermittent hypoxia and enhanced sympathetic activity increase the risk of cardiovascular disease and cognitive impairment in individuals with OSA (Brożyna-Tkaczyk et al., 2021). The mechanism by which OSA prompts cardiovascular diseases includes the increase in oxidative stress and activation of HIF-1α, both implicated in the modulation of the BK channel activity (Gabryelska et al., 2020; Orrù et al., 2020; Chen et al., 2021). CPAP, the most frequent treatment for OSA, improves the quality of life, decreased blood pressure to normal levels, and caused a significant reduction in oxidative stress (Zhao and Mehra, 2017; Baran et al., 2021).

Leukocytes from patients with obstructive sleep apnea-hypopnea syndrome (OSAHS) showed a decrease in the mRNA expression of the BK channel. When patients were exposed to CPAP therapy, it was observed a significant increase in the expression of the BK β1 subunit, which was paralleled with the adjustment of blood O_2_ tension. The authors reported a relation between oxygenation level, arterial tension, and BK β1 subunit expression. Considering the effect of CPAP in the BK subunit expression, it is suggested that BK channels contribute to vascular dysregulation in OSAHS (Navarro-Antolín et al., 2009; Caballero-Eraso et al., 2019). Endothelial dysfunction is an early clinical marker of atherosclerosis, and a risk marker for cardiovascular diseases. Patients with OSA showed endothelial dysfunction, correlated with a decrease in the expression of the BK β1 subunit, which was recovered after CPAP. An improvement in endothelial function was also observed after CPAP treatment, diminishing the cardiovascular risk (Caballero-Eraso et al., 2019). However, the CPAP treatment has not been demonstrated to provoke a significant blood pressure lowering effect in patients with OSA and nocturnal hypertension (Chen et al., 2020). These findings suggest that it is necessary to investigate the exact pathophysiological mechanisms involved in hypertension and cardiovascular risk associated with OSA.

## Conclusion and Perspective

Hypoxia, mediated by OSA, promotes a reduction in the BK β1 subunit expression that could induce a decrease in the BK channel Ca^2+^ sensitivity, maintaining membrane depolarization, and triggering the activation of L-type Ca^2+^ channels. The opening of these channels will produce a rise in [Ca^2+^]_i_. The induced response promotes the development of pathologies like hypertension, heart attack, and stroke, all of which are associated with hypoxia. Considering the described processes associated with hypoxic response, the mechanisms involved in OSA-derived hypoxia may be related to the increase in oxidative stress. However, there is no evidence to confirm that hypothesis, and further studies are necessary. In addition, there is no information about the effect of OSA in the expression of α or other β subunits. It could be interesting to carry on a deep study of the effect of OSA in the expression of the different BK subunits, as well as the possible role of other ion channels in the modulation of the BK channel activity. Moreover, exploration of other processes induced by hypoxia as HO-2 or HIF-1α regulation are important to unveil the complete mechanism involved in BK channel regulation by OSA, which could be considered a therapeutic approach to treat diseases derived from that disorder.

## Author Contributions

SO, LO, AA-P, FH, IC, and YT wrote the manuscript. All authors contributed to the article and approved the submitted version.

## Conflict of Interest

The authors declare that the research was conducted in the absence of any commercial or financial relationships that could be construed as a potential conflict of interest.

## Publisher’s Note

All claims expressed in this article are solely those of the authors and do not necessarily represent those of their affiliated organizations, or those of the publisher, the editors and the reviewers. Any product that may be evaluated in this article, or claim that may be made by its manufacturer, is not guaranteed or endorsed by the publisher.
